# Book Reviews

**Published:** 1901-07

**Authors:** 


A Treatise on Appendicitis. By George Ryerson Fowler, M. D., Professor of Surgery in the New York Polyclinic; Examiner in Surgery, New York State Medical Examining Board; Surgeon to the Methodist-Episcopal Hospital; Surgeon-in-Chief to the Brooklyn Hospital, etc. Octavo, pages 235. Illustrated. Second edition, revised and enlarged. Philadelphia and London: J. B. Lippincott Co. 1900. [Price, $2.50.]

The last word has not been said as yet upon this most interesting and treacherous malady. It is because it is often so insidious and treacherous, that it becomes necessary to discuss it so frequently from different standpoints. Hence, the profession of medicine welcomes every monograph from authoritative hands that will throw additional light upon the screen, or aid in solving the many different problems that pertain to appendicitis.

This author is such a writer; he has had much experience at the bedside and operating-table, and, moreover, states his facts and opinions clearly and directly. The clinical pictures of the disease herein drawn are most instructive and helpful; and, besides, the section on differential diagnosis is one of the best we have seen. Complications, too, are most intelligently detailed, the infectious nature of the malady fully set forth and its bacteriology amply described.

In considering the nonoperative treatment of appendicitis, Fowler's opening paragraph is significant and really states the whole argument in a kernel. He says: "The absolute rejection of the operative treatment of appendicitis is only justifiable under circumstances where the consent of the patient, or those responsible for him, cannot be obtained, or when he is placed under conditions such as to prohibit the employment of proper surgical aid." It has taken a good while and some sad experiences to bring the profession to recognise, not to say admit, that appendicitis is a surgical disease, pure and simple, and that medicine, represented by drugs, has very little place in its management.

The operative treatment of appendicitis, as set forth by Fowler, is everything that could be desired. He considers the subject from every scientific viewpoint that has any practical bearing on the patient. His preparations are most complete, his execution is surgically artistic in the extreme, his after-treatment and dealing with post-operative complications embrace every known method of value. The illustrations are among the best, and reflect great credit upon the artist. Pictures can be made of great use in surgery, if they are accurate—they mislead greatly if they are not so. The author of this work is so artistic a surgeon that he could not admit to his book an inartistic or misleading illustration.

Many authors have written upon appendicitis; some excel in one direction, some in another. Each adds something to the general fund of knowledge. It has remained for Fowler to contribute so considerably toward the enlightenment of the profession on this subject, that his book easily takes rank in the forefront.

---

Progressive Medicine. Volume I., 1901. A quarterly digest of advances, discoveries and improvements in the medical and surgical sciences. Edited by Hobart Amory Hare, M. D., Professor of Therapeutics and Materia Medica in the Jefferson Medical College of Philadelphia. Octavo, bound in cloth, 430 pages, 11 illustrations. Philadelphia and New York: Lea Brothers & Co. 1901. [Per annum, in four cloth bound volumes, $10 00.]

The contents of this volume comprise sections on surgery of the head, neck and chest, prepared by J. Chalmers DaCosta; on infectious diseases, including acute rheumatism, croupous pneumonia and influenza, prepared by Frederick A. Packard; on diseases of children, prepared by Floyd M. Crandall; on pathology, prepared by Ludwig Hektoen; on laryngology and rhinology, prepared by A. Logan Turner; and on otology, prepared by Robert L. Randolph. These writers and compilers are well and favorably known to the profession. It is unlikely that they would permit their names to be attached to unworthy literature. The subjects dealt with, too, are of an eminently practical character; hence, they appeal to the practising physician and surgeon in city or country. The popularity of this form of literature appears to be constantly increasing. There is little wonder for this, because in this period of bustle and whirl the busy physician needs condensed chapters in short meter. In this series he gets them and they are of the best.

---

Uterine Tumors; Their Pathology and Treatment. By W. Roger Williams, Fellow of the Royal College of Surgeons. Duodecimo, pp. 375. Illustrated. New York: William Wood & Co. 1901. [Price, $3.00 net.]

The great interest attaching to the study of tumors of the uterus at the present day, makes any new light turned in upon their pathology or treatment eagerly sought after by gynecological surgeons all over the world. The preface to this book is one of the best we ever saw; it says, after quoting Herbert Spencer, that our medical science lacks the eye of philosophy; that it is a tangle of disjointed facts; that in the words of Reman, it is "not unlike a magnificent library turned upside down. Nearly everything is there, but in such a confused, pell-mell, and thoroughly unclassified state, that it might almost as well not be there." Further on he quotes Buckle as follows: "Our scientists display an inordinate respect for experiments, an undue love of minute details, and a disposition to over-rate the invention of new instruments, and the discoverers of new, but often insignificant facts. . . . . We are in the predicament that our facts have outstripped our knowledge, and are now encumbering our march. The publications of our scientific institutions, and of our scientific authors, overflow with minute and countless details, which perplex the judgment, and which no memory can retain. In vain do we demand that they should be generalised and reduced into order. Instead of that the heap continues to swell."

The author of this monograph thinks that such being the condition of the science of medicine, the true task for authors now is generalisation and reduction into order of the masses of chaotic facts, that have been heaped upon the profession with such ruthlessness. He announces that he presents this book in the spirit foreshadowed in the foregoing abstract of his preface. How well he has succeeded only a careful reading of the work can determine. Every one interested will surely obtain it, because it is a contribution to the literature of a subject of transcendent importance, is well planned and interestingly written by a master of the topic; and, moreover, is easily the best recent monograph relating to it that has been offered to the medical profession.

---

Obstetric and Gynecologic Nursing. By E. P. Davis, A. M., M. D., Professor of Obstetrics in Jefferson Medical College and Philadelphia Polyclinic. Duodecimo, 402 pages, fully illustrated. Philadelphia and London. W. B. Saunders & Co. 1901. [Price, $1.75 net.]

While every nurse should be instructed well in the ground work or principles of the nursing profession, if we may be permitted to call it such, yet the obstetric nurse in particular needs special training to fit her for the responsible duties she is about to undertake. This, too, will apply in a large measure to the gynecologic nurse, whose duties are often as distinctly special, as are those of the gynecologist himself.

As there are specialists among physicians or in the medical profession, so, too, are there specialists among nurses, hence it is most fitting and timely that special works on the several specialties pertaining to nursing should be written and delivered to the profession of nursing. The book before us is prepared by an experienced instructor, one who well knows the needs of nurses in the special branches of obstetrics and gynecology. In it one will find the essentials of the subjects treated of, told in an interesting manner. It instructs the nurse in regard to natural pregnancy, as well as the signs and accidents of disease that may occur during gestation. There is also careful instruction given concerning the care of the woman during labor, and the needs of the child during the early weeks of its life. The principles of asepsis and antisepsis are admirably discussed, so that the gynecologic nurse may be well informed as to those important topics.

An appendix contains an excellent dietary and a section regardng the preparation of surgical supplies. It is a book that should be in the hands of every one who may be called upon to do obstetric or gynecologic nursing. Its teachings are sound, its instructions are plain, and it is the most complete work of its kind that has yet been published.

---

A Reference Handbook of the Medical Sciences. Embracing the Entire Range of Scientific and Practical Medicine and Allied Science. By various writers. A new edition, completely revised and rewritten. Edited by Albert H. Buck, M. D., New York City. Volume II. Bla–Chl. Illustrated by numerous chromo-lithographs and 765 half-tone and wood engravings. Nine volumes, imperial 8vo. New York: William Wood & Co. 1901. [Price, muslin, $6.00 per volume; leather, $7.00 per volume; half-morocco, $8.00 per volume.]

The second volume of this elaborate series is a most satisfying book, whether viewed from its scientific importance or from the artistic quality of its engravings. The article on the blood is an excellent exposition of our present knowledge concerning the pathology and methods of studying the life-giving current. It is well worth the price of the volume, to say nothing of the wealth of material the book contains besides, this one paper. Another contribution of great importance is that on the brain, which is exhaustive in character, as well as scientific in its manner of presentation. Medical officers in the military service will be much interested in the article on camp diseases, which is well written and modern in its material. And so we might go on pointing out subjects of importance that are superbly considered in this volume; but we could not be expected to review the book in the ordinary sense of the word, for it would take up the entire space of this issue of the magazine, index and all, to give critical analysis of each article.

The Buffalo contributors to this number are Herbert M. Hill, Abram T. Kerr (now of Ithaca) and Julius Pohlman—names well-known in the literature of medicine, and competent men to perform their full share in making this volume a credit to the profession.

Some of the plates are superb, among which may be mentioned No. xxi., illustrating carcinoma of the skin, also Councilman's photomicrographs (plate xx.) showing some of the characteristics of carcinoma. The subscribers to this series need not feel anxious about their investment—it is one of the best that can be made in a reference work of medicine.

---

Diseases of the Nose and Throat. By D. Braden Kyle, M. D., Clinical Professor of Laryngology and Rhinology, Jefferson Medical College, Philadelphia; Consulting Laryngologist, Rhinologist and Otologist, St. Agnes' Hospital. Second edition, revised. Octavo, 646 pages. Over 150 illustrations and 6 lithographic plates. Philadelphia and London: W. B. Saunders & Co. 1901. [Cloth, $4.00 net.]

This excellent treatise, the first edition of which was reviewed in the Journal, January, 1900, has appeared again within an unusually short time. This, alone, indicates the appreciation the work received at the hands of specialists, since, in the nature of things, they must be the largest purchasers. There is, however, much in the book that will aid and instruct the general practitioner of medicine. It is he, often, who will treat the simpler forms of diseases of the nose and throat; or will minister to others in their initial stages, hence he must, perforce, inform himself as to methods of diagnosis and become more or less skilful in the instrumentation of these passages.

The author is strong in the pathological handling of his subject, and for this the specialist will be thankful, though it may be too intricate for the untrained in technical knowledge of the microscope. Diseases of the upper air tract are so often the cause of reflex symptoms below, which mislead in management, that it behooves every physician to be on the alert in these matters, lest grievous errors be committed in the name of medical treatment. Kyle's book is one that deserves careful study, as it easily takes rank among the best works on laryngology yet printed. This edition does not differ materially from the last, but some errors have been corrected and in general matters it has been brought forward to the immediate present. Its illustrations deserve special commendation.

